# Supplementary material for: The Stroop effect involves an excitatory–inhibitory fronto-cerebellar loop
Source: Nat Commun. 2023 Jan 11;14:27. doi: 10.1038/s41467-022-35397-w (PMC9834394; doi:10.1038/s41467-022-35397-w)
Supplement: Supplementary file 1 — Supplementary Information [file 41467_2022_35397_MOESM1_ESM.pdf]

Supplementary Information

Title: The Stroop effect involves an excitatory–inhibitory fronto–cerebellar loop

Authors: Moe Okayasu,[1] Tensei Inukai,[1] Daiki Tanaka,[1] Kaho Tsumura,[1]
Reiko Shintaki,[1] Masaki Takeda,[2] Kiyoshi Nakahara,[2] Koji Jimura[1,2,3]

Affiliations: [1] Department of Biosciences and Informatics,
Keio University
[2] Research Center for Brain Communication,
Kochi University of Technology
[3] Department of Informatics,
Gunma University

Correspondence should be addressed to:
Koji Jimura, Ph.D.
Department of Informatics,
Gunma University,
4-2 Aramaki-cho Maebashi,
371-8510, Japan
Email: jimura@gunma-u.ac.jp
Phone: +81-27-220-7579

Supplementary Figures

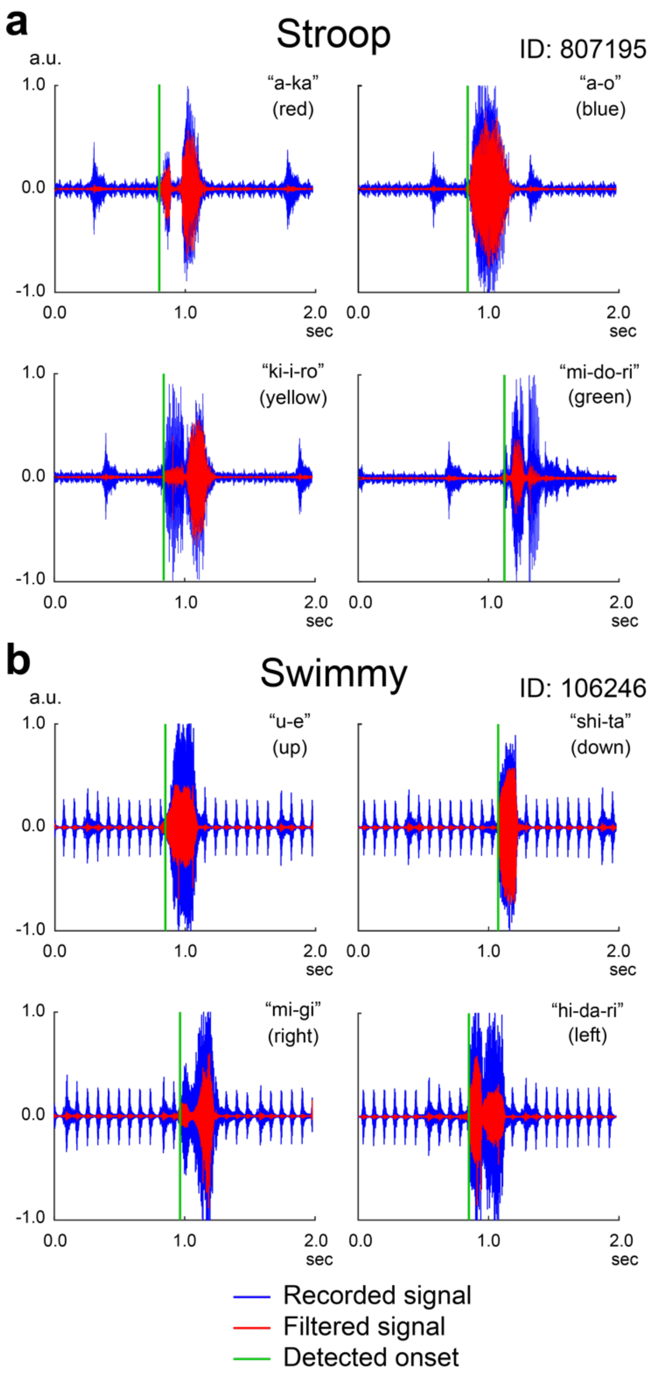

Supplementary Figure 1. Detection of vocal onsets.
Vocal data samples for the Stroop (**a**) and Swimmy (**b**) tasks. Horizontal and vertical axes indicate time from the onset of the trial and magnitudes of the vocal signals, respectively. Recorded signals (blue) were filtered, and then when the filtered data (red) exceeded a threshold, the onsets of the vocal response (green) were identified.

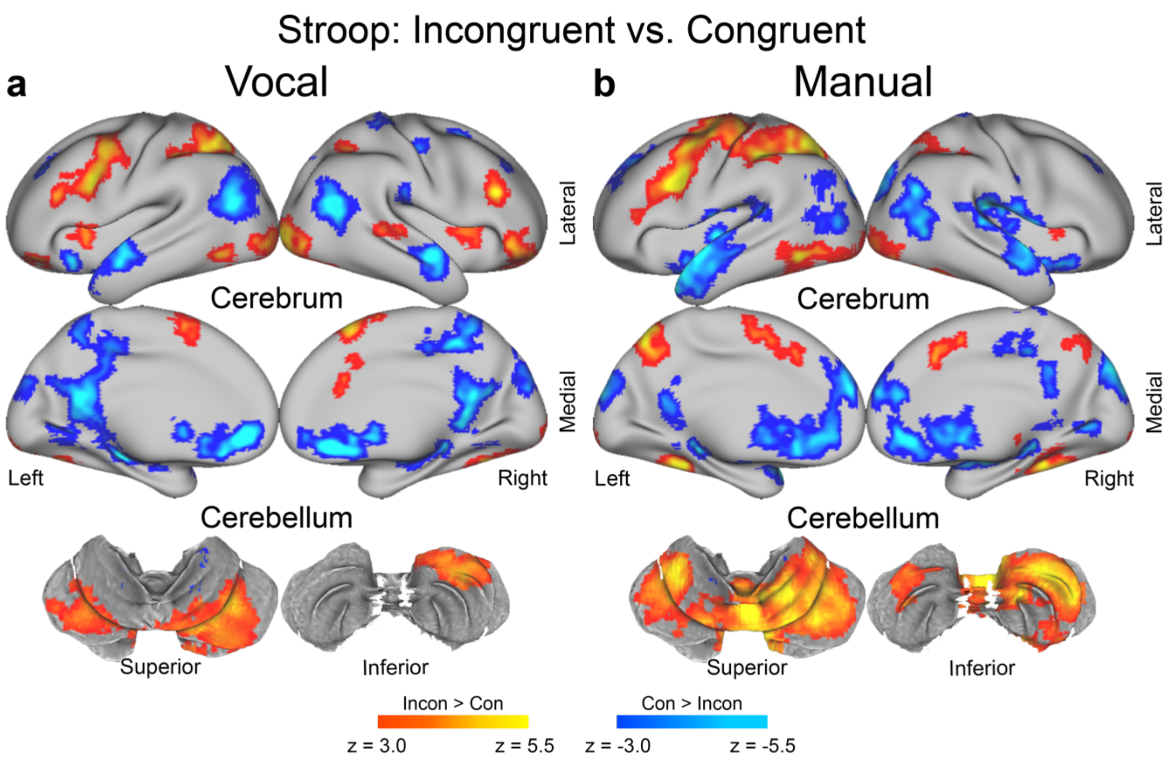

Supplementary Figure 2. The Stroop effect involves common lateralized cerebral and cerebellar regions in the vocal and manual response conditions.
Statistical activation maps for a signal increase or decrease in the contrast between incongruent and congruent trials for the Stroop task are separately shown for the vocal response condition (**a**) and manual response condition (**b**). Formats are similar to those in Fig. 3a.

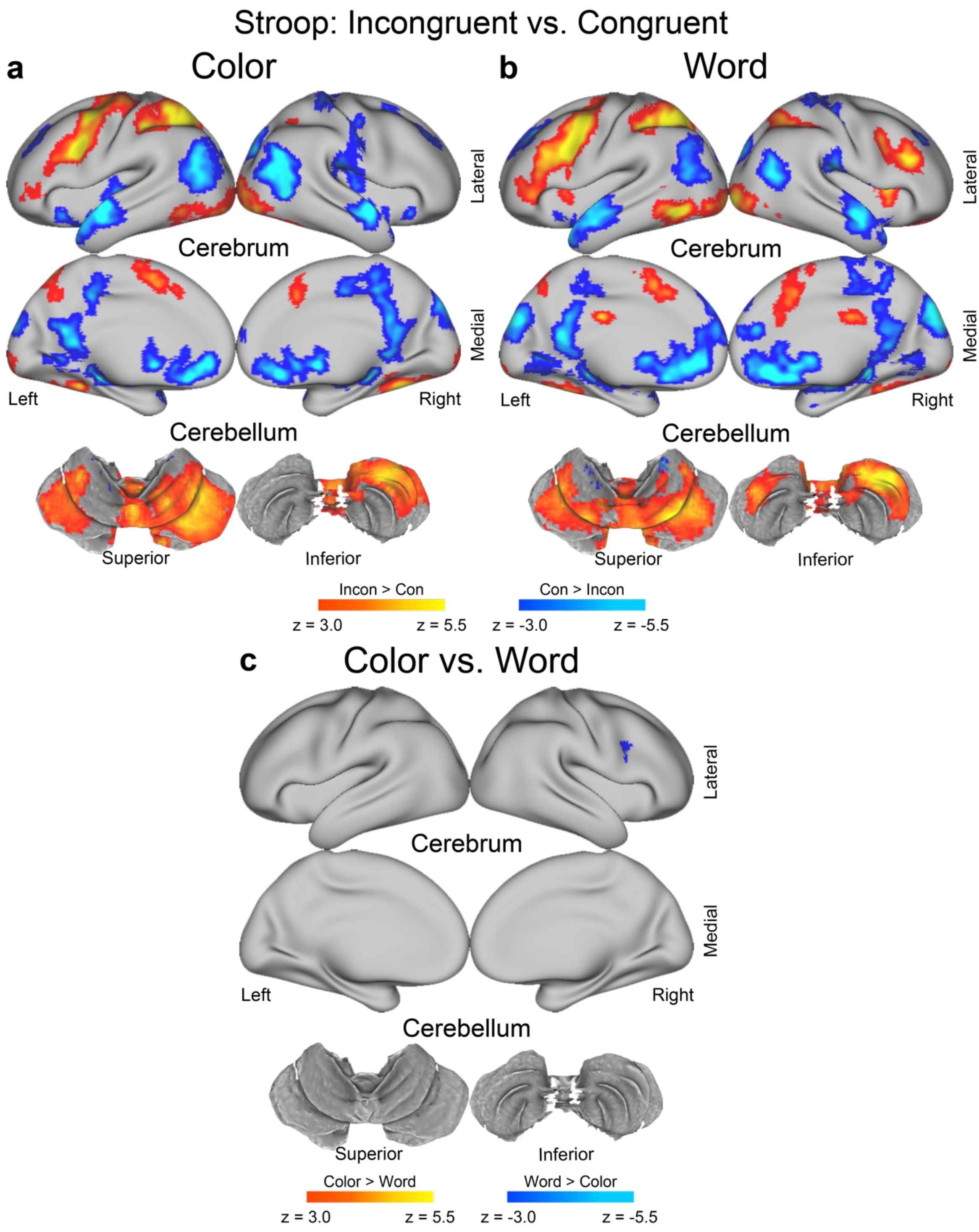

Supplementary Figure 3. The interference effect in the color and word tasks involve common cerebral and cerebellar regions.
Statistical activation maps for a signal increase or decrease in the contrast between incongruent and congruent trials in the Stroop task are separately shown for the color task (**a**) and the word task (**b**). **c**, Statistical maps showing differential brain activity the interference effect between the color and word tasks. Formats are similar to those in Fig. 3.

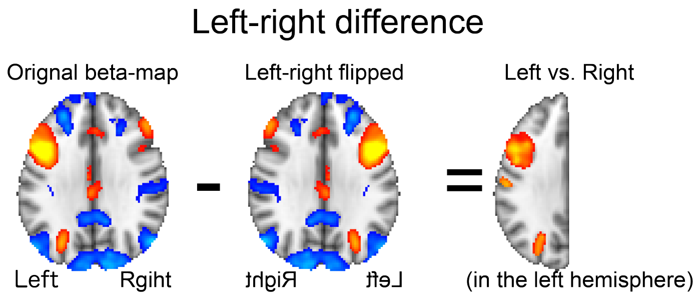

Supplementary Figure 4. Analysis procedure for exploration of lateralized activity.
Contrast maps were flipped along the X axis (left-right), then subtracted from the original non-flipped maps before being entered into voxel-wise group-level analyses in the left hemisphere.

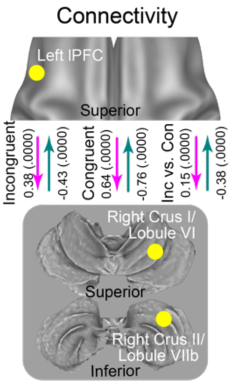

Supplementary Figure 5. DCM analysis for the Stoop task.

The left lPFC ROI was defined based on a meta-analysis map of cognitive control. Statistical procedures and formats are similar to those in Fig 3c.

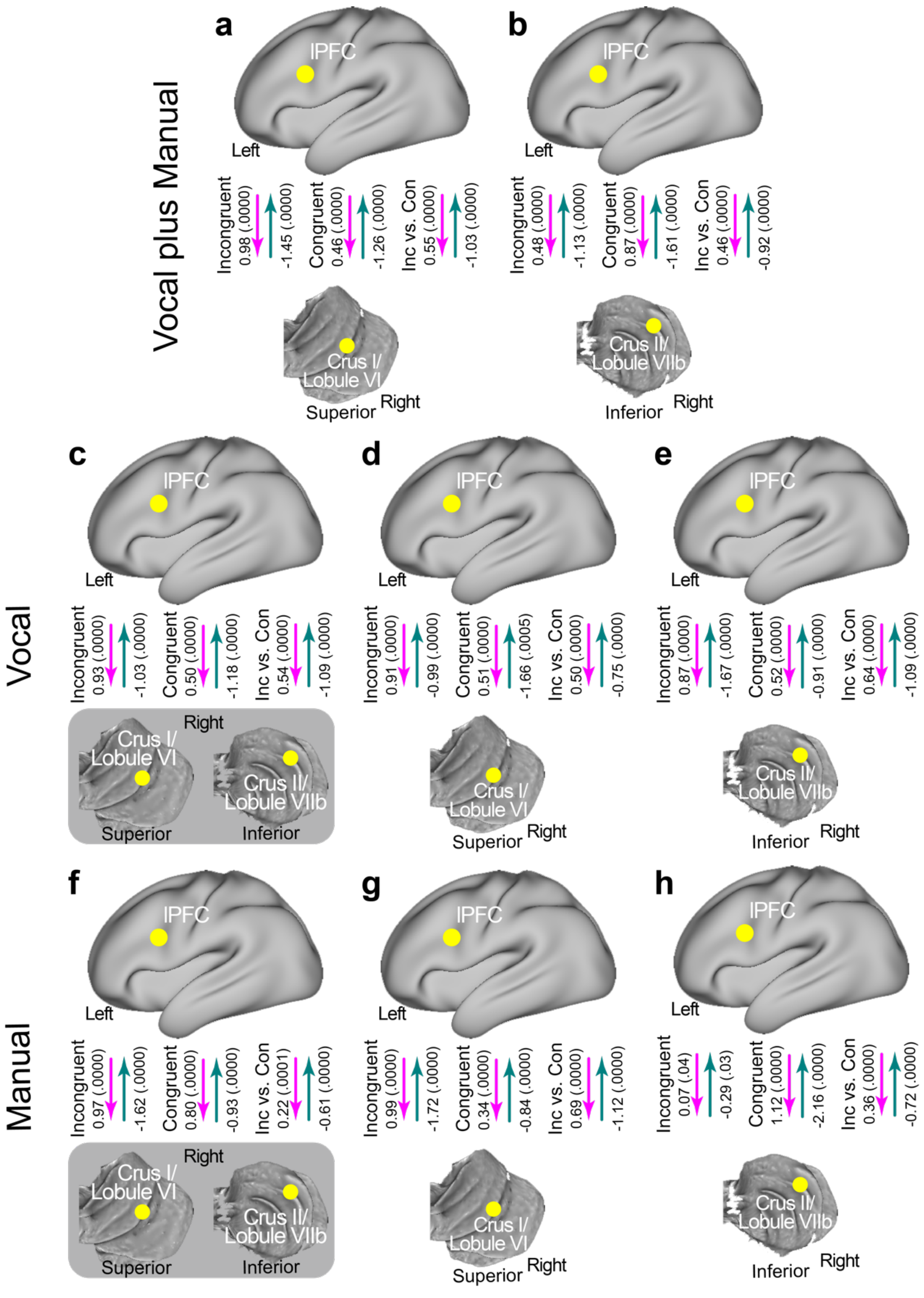

Supplementary Figure 6. Task-related effective connectivity between the lPFC and cerebellar regions during the Stroop task.

Connectivity with the lPFC was estimated separately for the crus I/lobule VI (**a**) and crus II/lobule VIIb (**b**). **c-h**, The vocal and manual response conditions were analyzed separately. **C-e**: Vocal response condition. **f-h**, Manual response condition. Connectivity between the lPFC and the two cerebellar regions (**c**/**f**), between the lPFC and crus I/lobule VI (**d**/**g**), and between the lPFC and crus II/lobule VIIb (**e**/**h**). Statistical procedures and formats are similar to those in Fig. 3c.

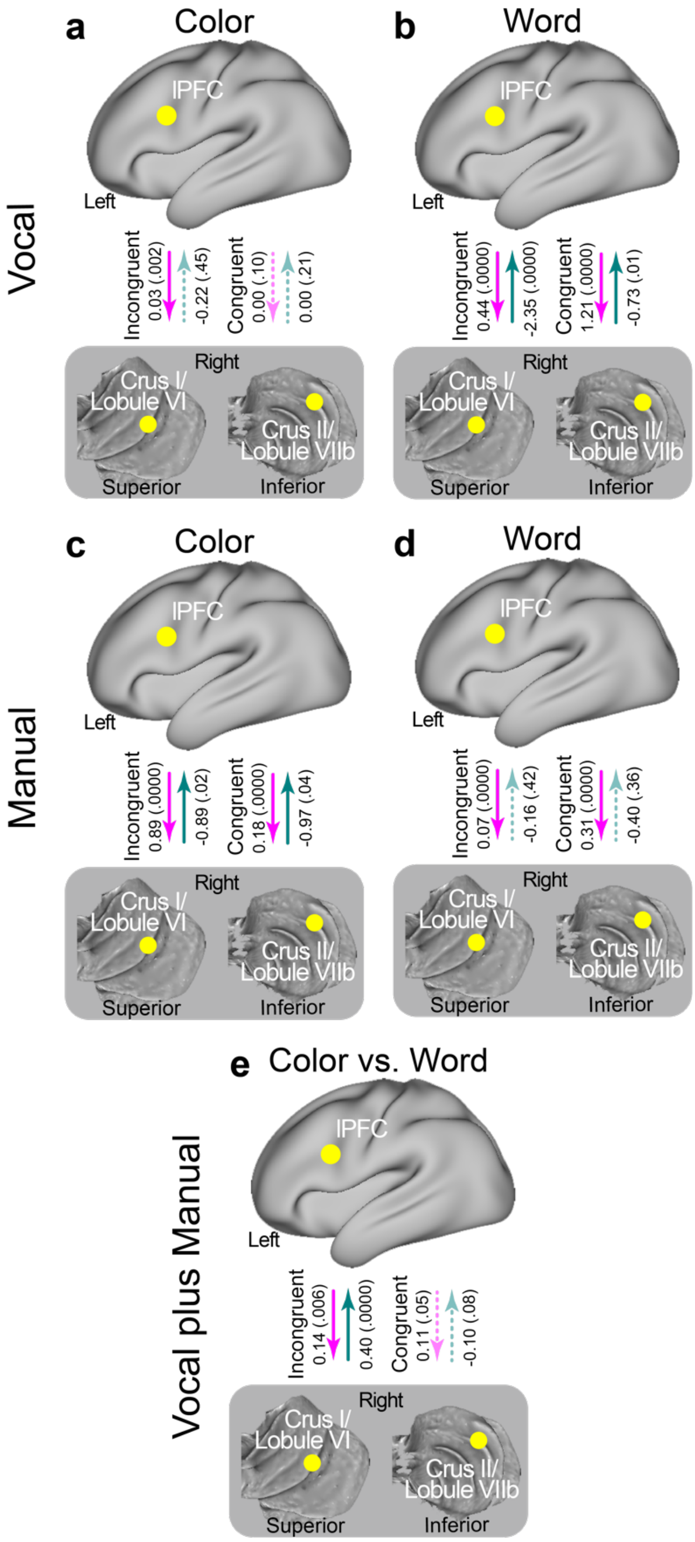

Supplementary Figure 7. Task-related effective connectivity for the color and word tasks in the Stroop tasks.

a/b, Vocal response condition; **c/d**, manual response condition; **a/c**, color task; **b/d**, word task. **E**, Connectivity strength was contrasted between the color and word tasks. Statistical procedures and formats are similar to those in Fig. 3c.

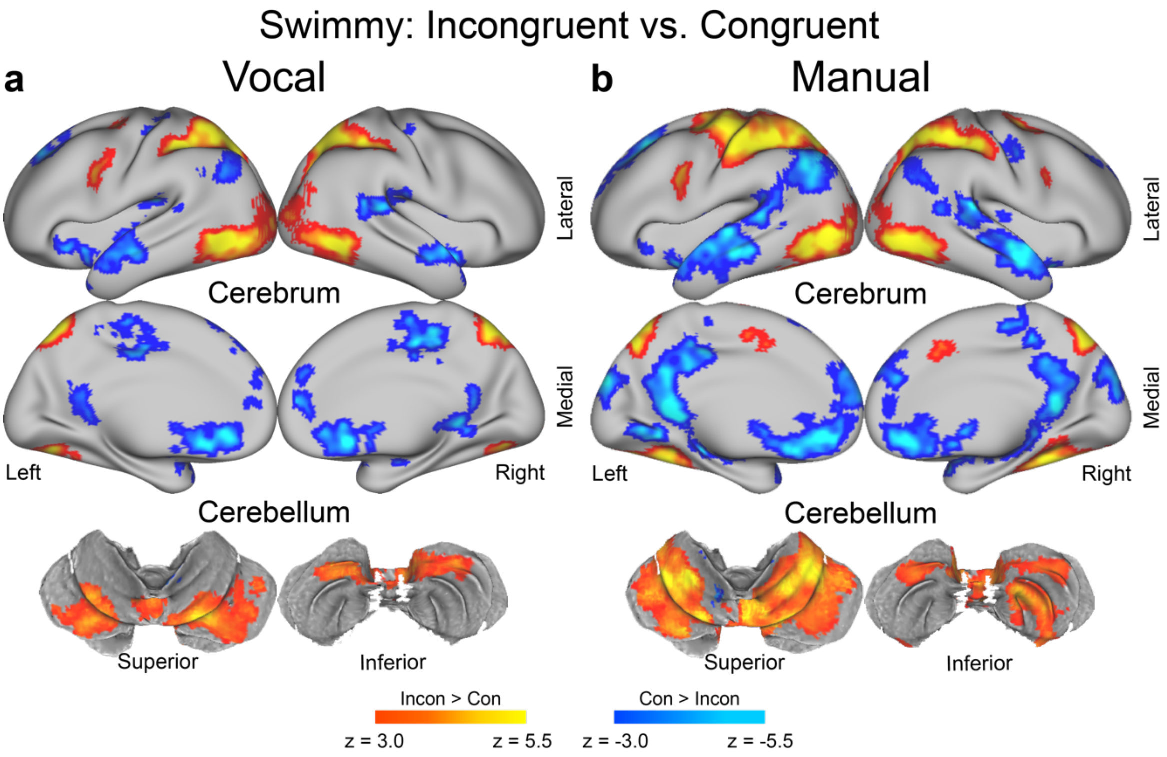

Supplementary Figure 8. The Swimmy effect involves common bilateral cerebral and cerebellar regions in the vocal and manual response conditions.
Statistical activation maps for a signal increase or decrease in the contrast between incongruent and congruent trials in the Swimmy task are shown separately for the vocal response condition (**a**) and the manual response condition (**b**). Formats are similar to those in Fig. 3.

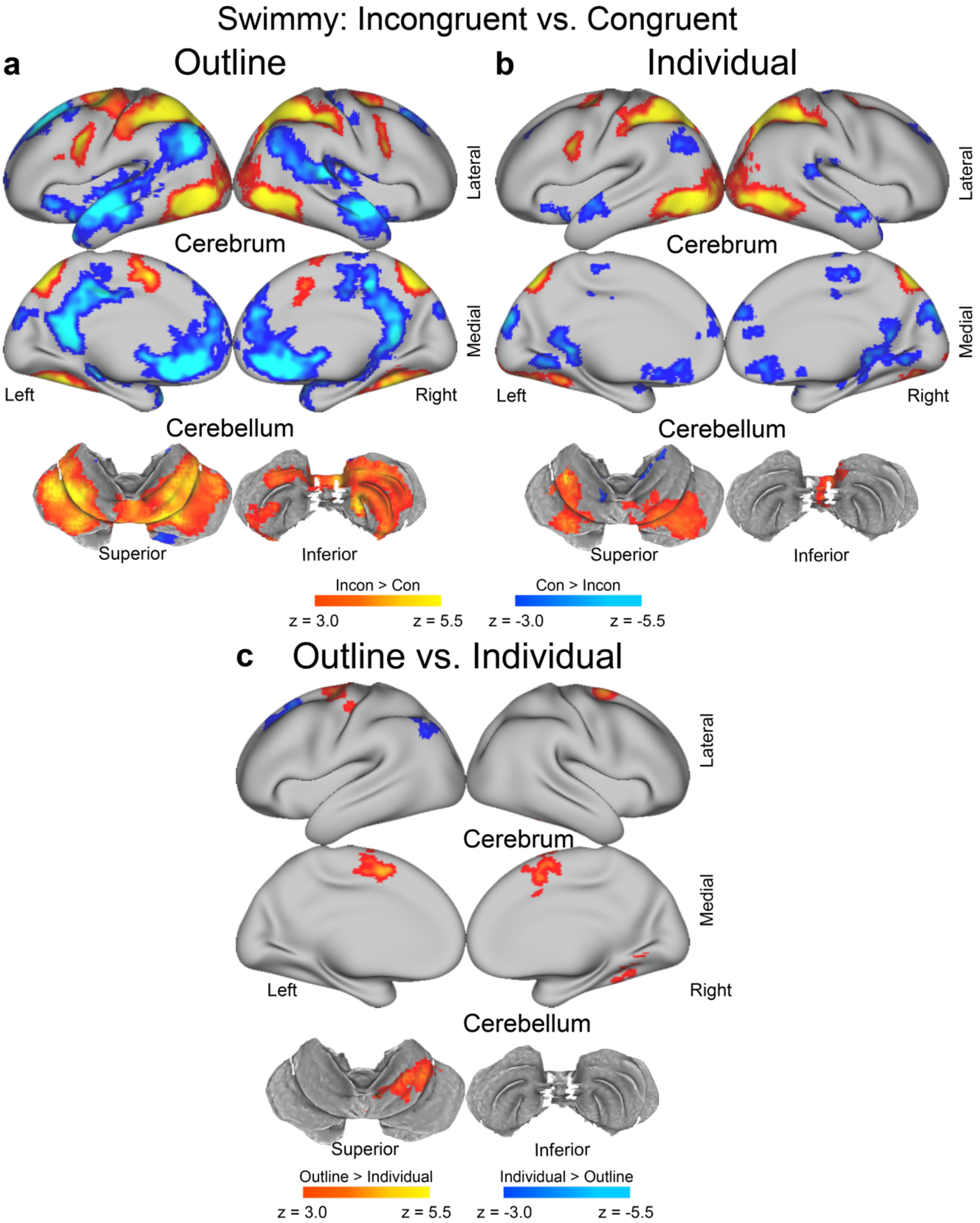

Supplementary Figure 9. The interference effect in the outline and individual tasks involve common cerebral and cerebellar regions.
Statistical activation maps for a signal increase or decrease in the contrast between incongruent and congruent trials in the Swimmy task are shown separately for the outline task (**a**) and the individual task (**b**). **c**, Statistical maps showing differential brain activity in the interference effect between the color and word tasks. Formats are similar to those in Fig. 3.

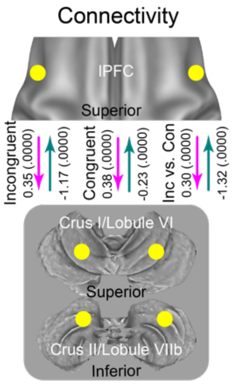

Supplementary Figure 10. DCM analysis for the Swimmy task.

The left lPFC ROI was defined based on a meta-analysis map of cognitive control. Statistical procedures and formats are similar to those in Fig 3c.

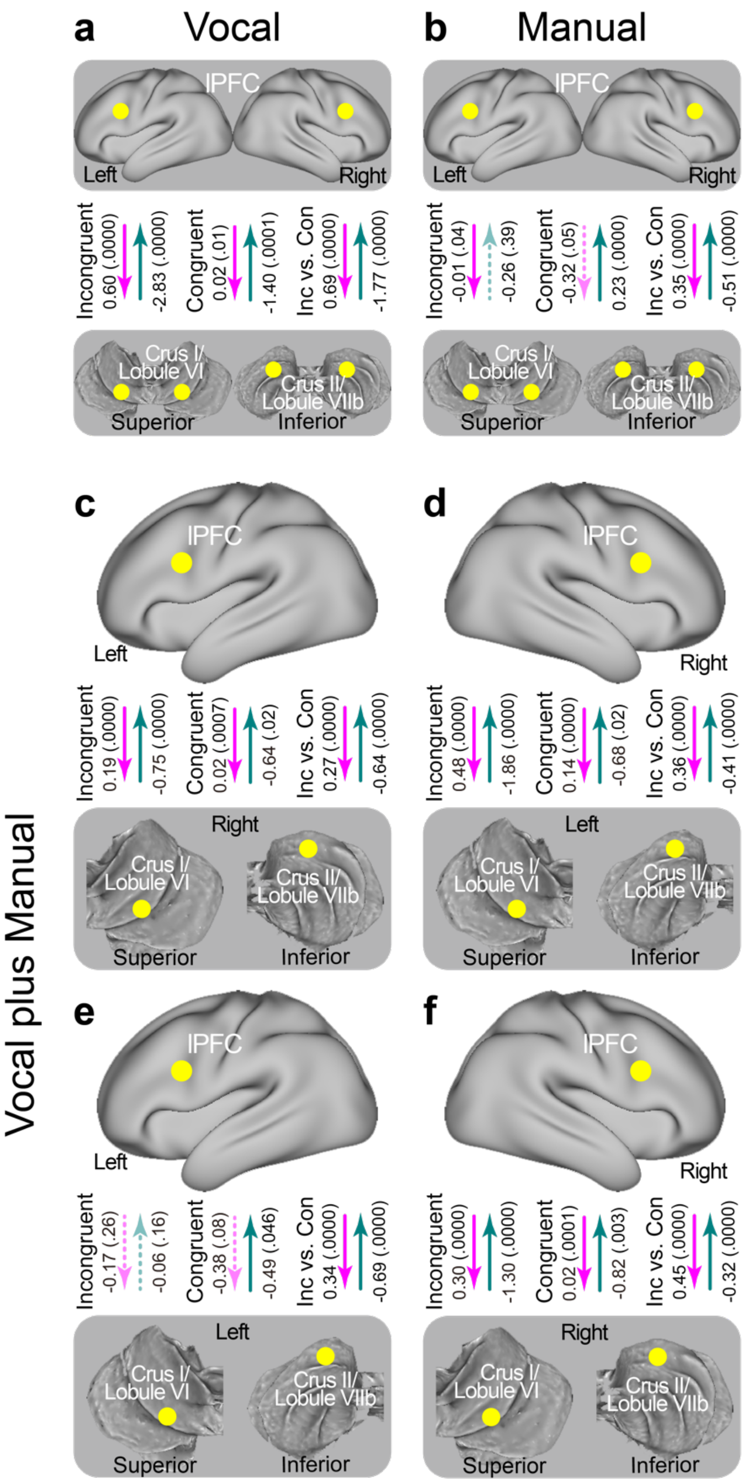

Supplementary Figure 11. Task-related effective connectivity between the lPFC and cerebellar regions during the Swimmy task.

Connectivity with the lPFC was estimated separately for the vocal response condition (**a**) and the manual response condition (**b**). **c-f**, Connectivity was estimated between the lPFC and cerebellum in either of the right or left hemispheres. **c**, Left lPFC and right cerebellum; **d**, right lPFC and left cerebellum; **e**, left lPFC and left cerebellum; **f**, right lPFC and right cerebellum. Statistical procedures and formats are similar to those in Fig 3c.

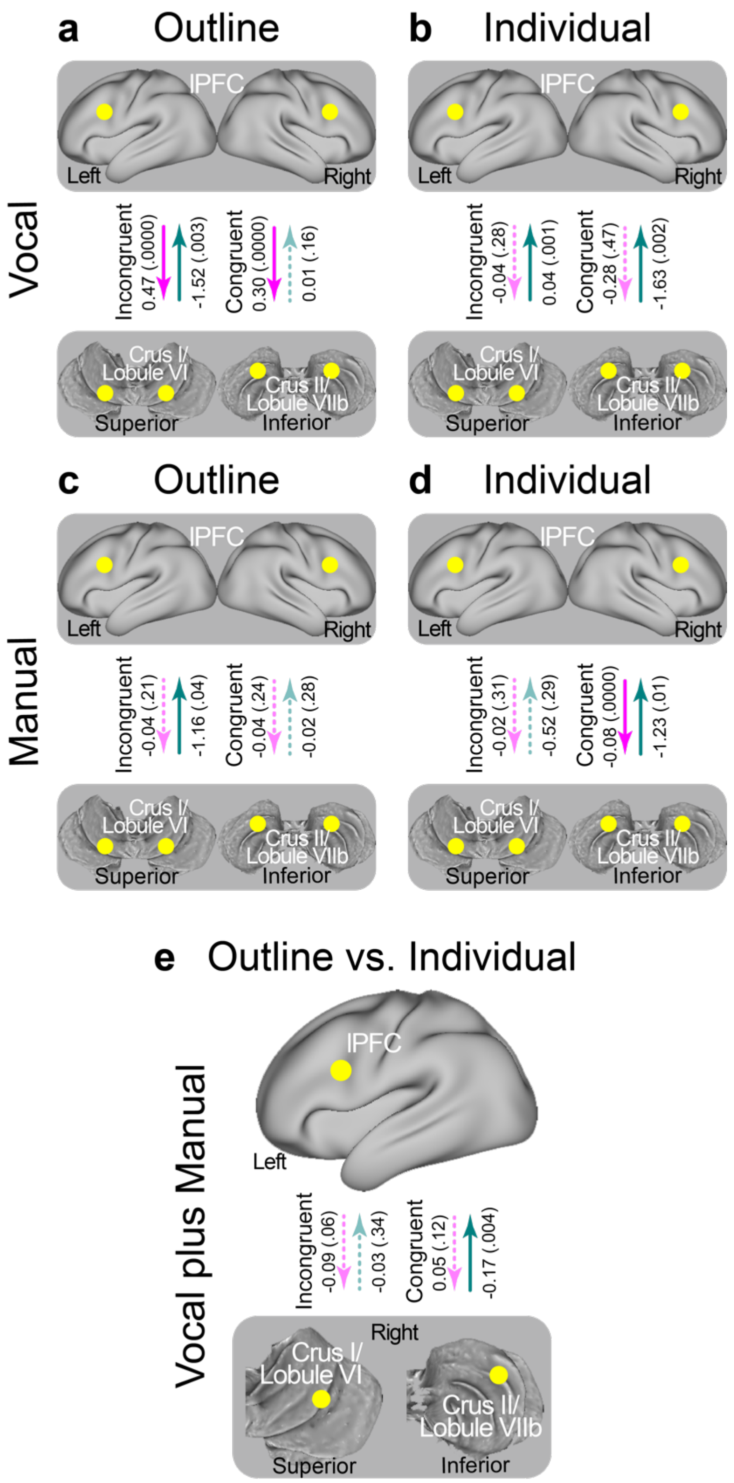

Supplementary Figure 12. Task-related effective connectivity during the outline and individual tasks in the Swimmy task.

a/b, Vocal response condition; **c/d**, manual response condition; **a/c**, outline task; **b/d**, individual task. **e**, Connectivity strength was contrasted between the outline and individual tasks. Statistical procedures and formats are similar to those in Fig. 3c.

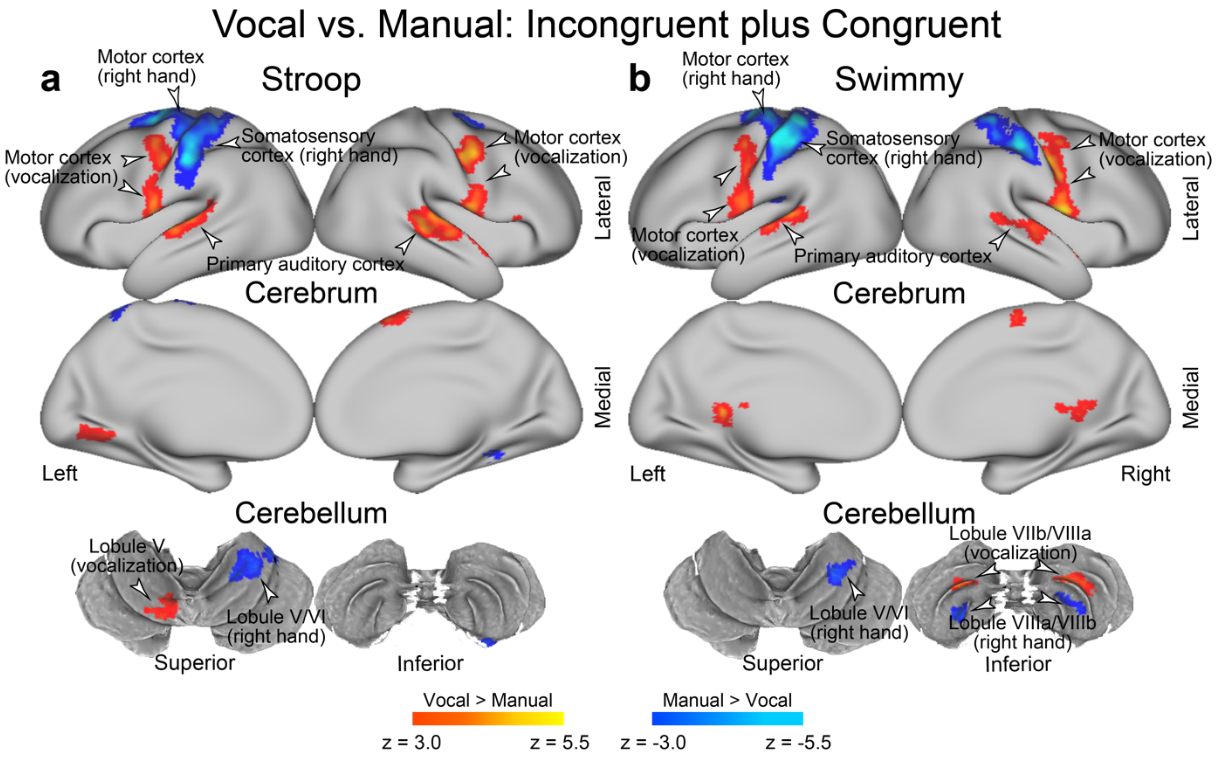

Supplementary Figure 13. Vocal and manual conditions involve distinct sensory and motor regions. Statistical activation maps for a signal increase or decrease in the contrast between vocal and manual response conditions. **a**, Stroop task; **b**, Swimmy task. The incongruent and congruent trials are collapsed. Formats are similar to those in Fig. 3.

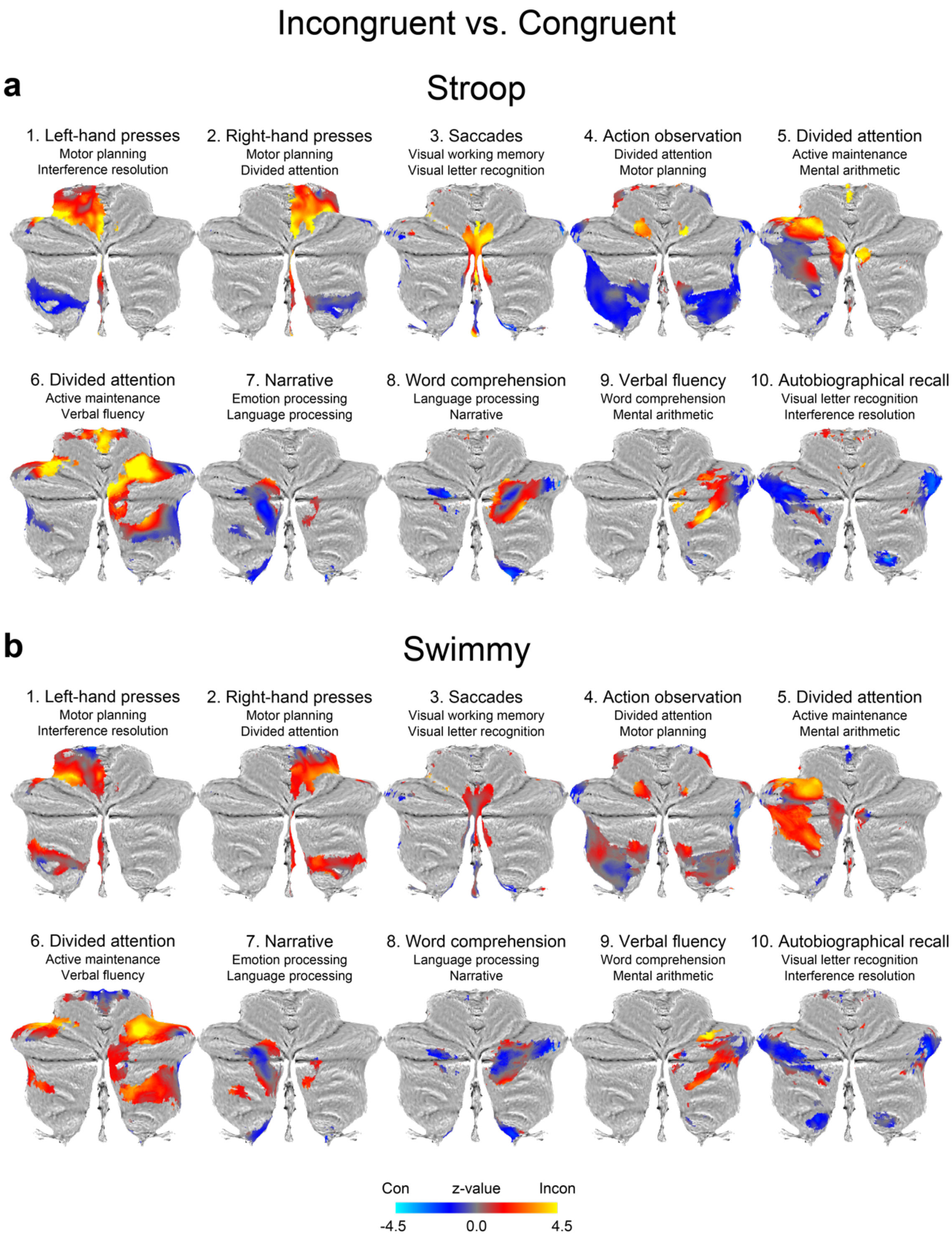

Supplementary Figure 14. Statistical z-values for the contrast of the incongruent vs. congruent trials are masked by cerebellar ROIs defined by functional parcellation in a previous study, and are then mapped onto 2D flat maps of the cerebellum. **a**, Stroop task; **b**, Swimmy task. Formats are similar to those in Fig. 6b.

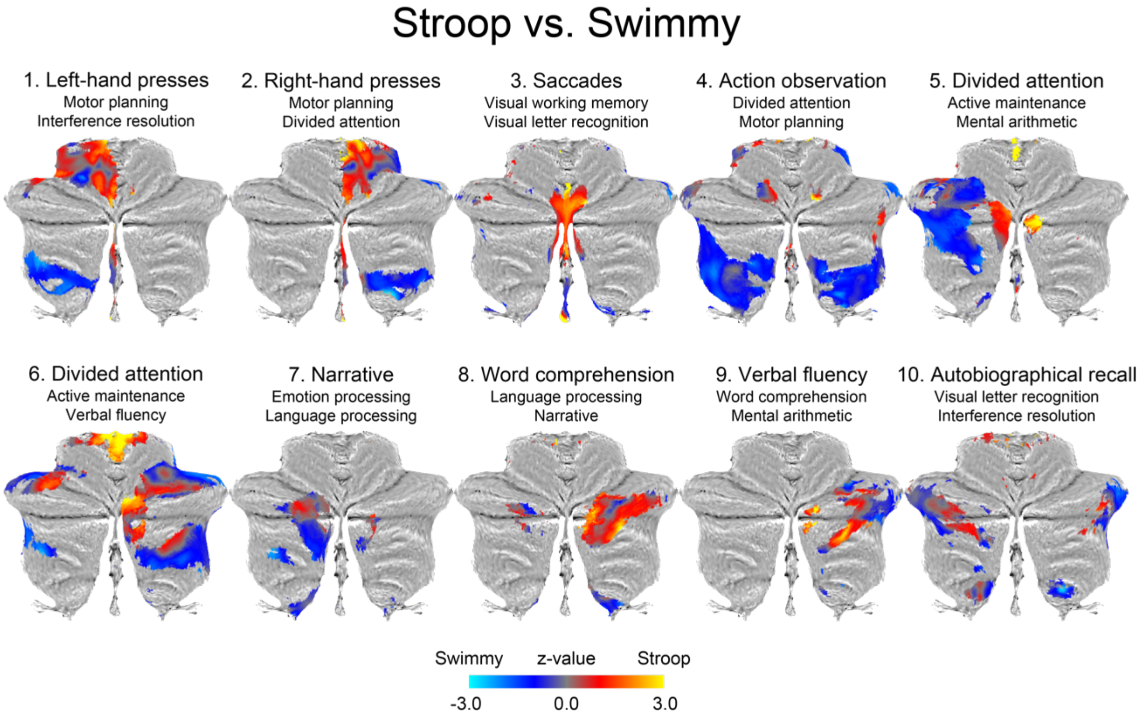

Supplementary Figure 15. Statistical z-values for the differential interference effect (incongruent vs. congruent) between the Stroop and Swimmy tasks are masked by cerebellar ROIs defined by functional parcellation in a previous study and are then mapped onto 2D flat maps of the cerebellum. Formats are similar to those in Fig. 6b.

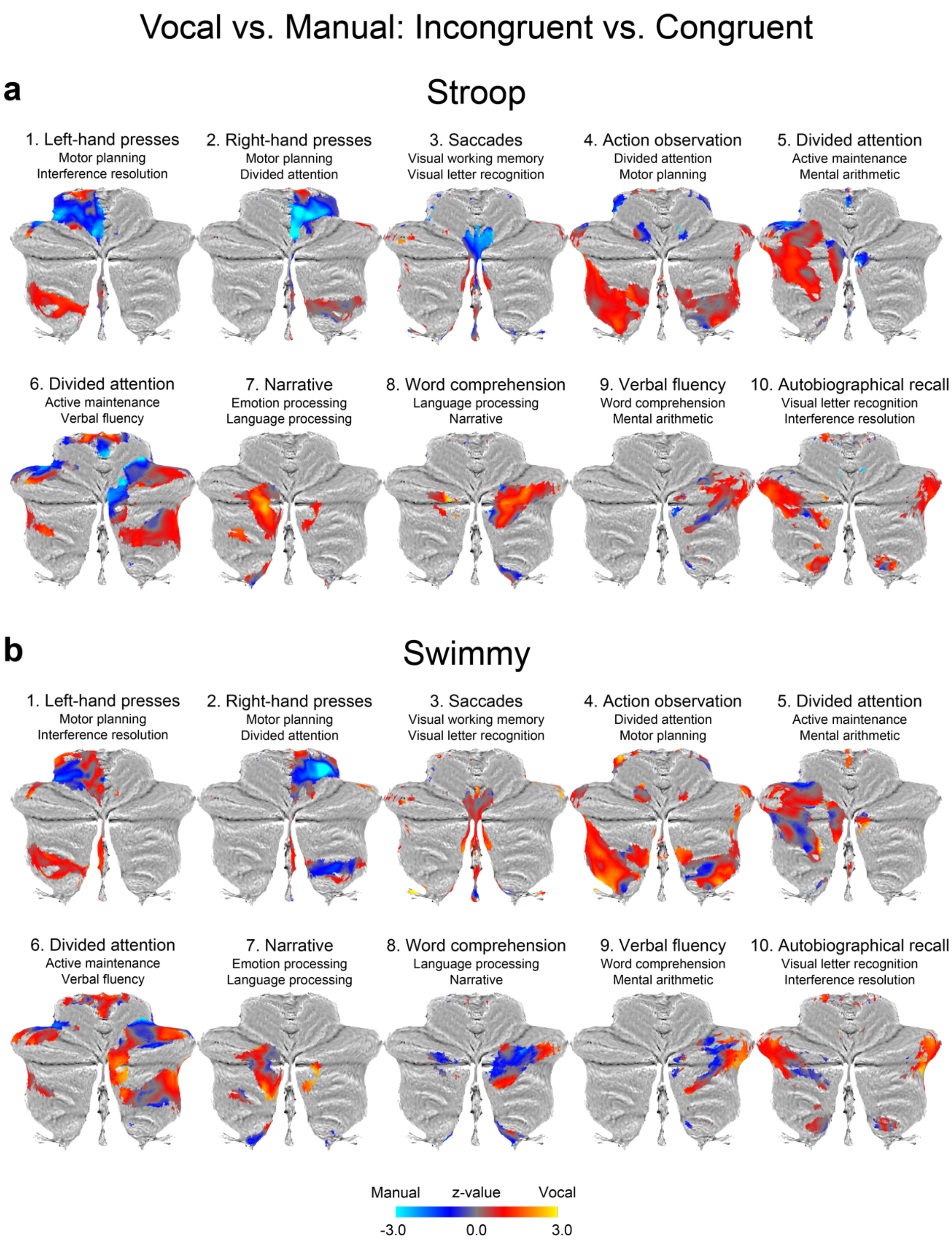

Supplementary Figure 16. Statistical z-values for the differential interference effect (incongruent vs. congruent) between the vocal and manual conditions are masked by cerebellar ROIs defined by functional parcellation in a previous study and are then mapped onto 2D flat maps of the cerebellum. **a**, Stroop task; **b**, Swimmy task. Formats are similar to those in Fig. 6b.

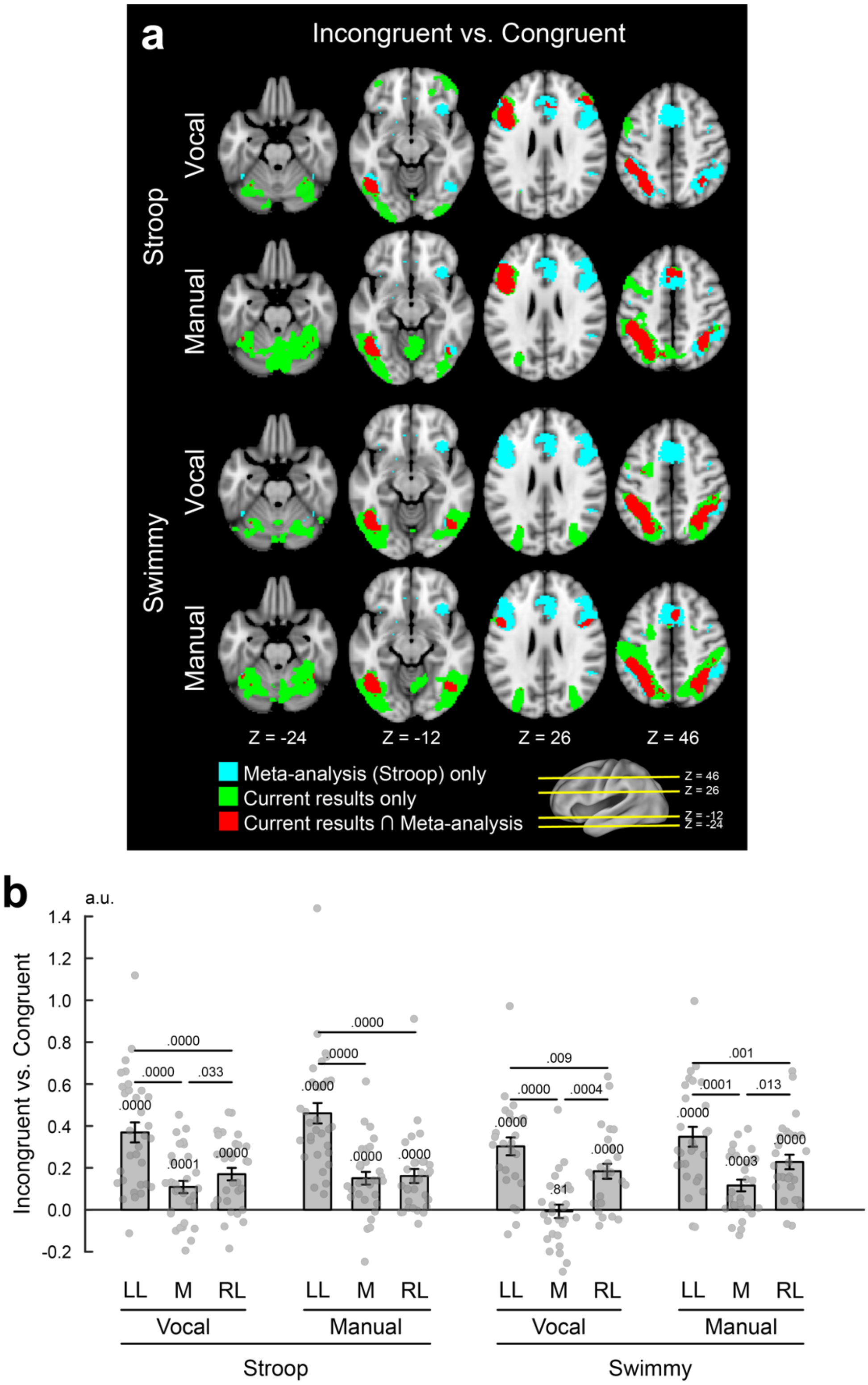

Supplementary Figure 17. Comparisons with meta-analysis data for vocal and manual conditions. **a**, Meta-analysis maps of the Stroop effect and current activation maps of interference effects (incongruent vs. congruent in the Stroop and Swimmy tasks) are overlaid on transverse sections of structural images separately for the vocal and manual conditions. Formats are similar to those in Fig. 7a. **b**, Regions-of-interest analysis of the Stroop and Swimmy tasks are shown separately for the vocal and manual response conditions. Statistical procedures and formats are similar to those in Fig. 7b. The sample size is identical to that in Fig 2a.

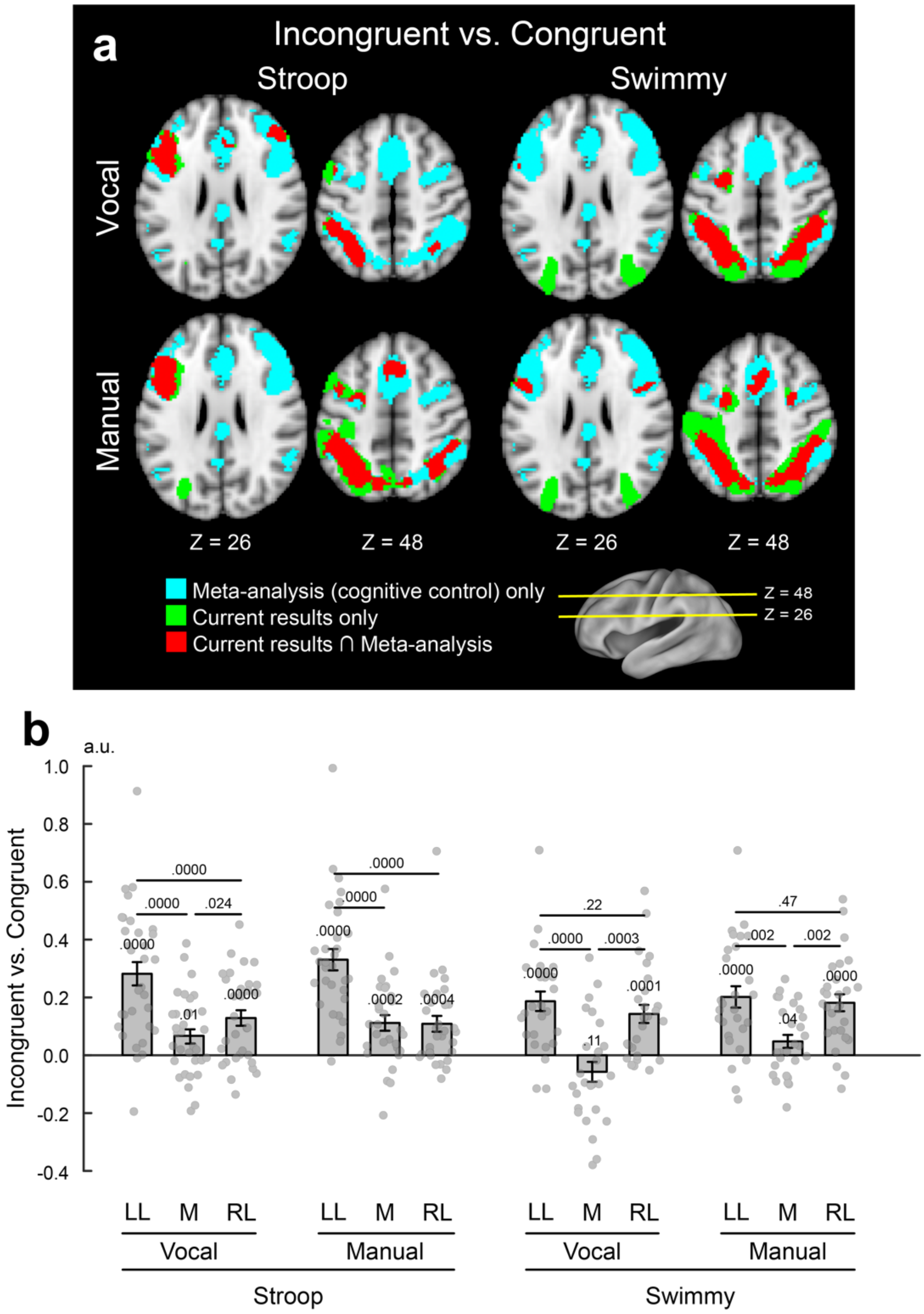

Supplementary Figure 18. Comparisons with meta-analysis data of cognitive control.
a, Meta-analysis maps of cognitive control and current activation maps of interference effects (incongruent vs. congruent) are overlaid on transverse sections of structural images. Vocal response condition (*top*); manual response condition (*bottom*); Stroop task (*left*); Swimmy task (*right*). Formats are similar to those in Fig. 7a. **b**, Region-of-interest analysis. Stroop task (*left*); Swimmy task (*right*). For each task, activity magnitudes were calculated separately for the vocal and manual response experiments. Statistical procedures and formats are similar to those in Fig. 7b. The sample size is identical to that in Fig 2a.

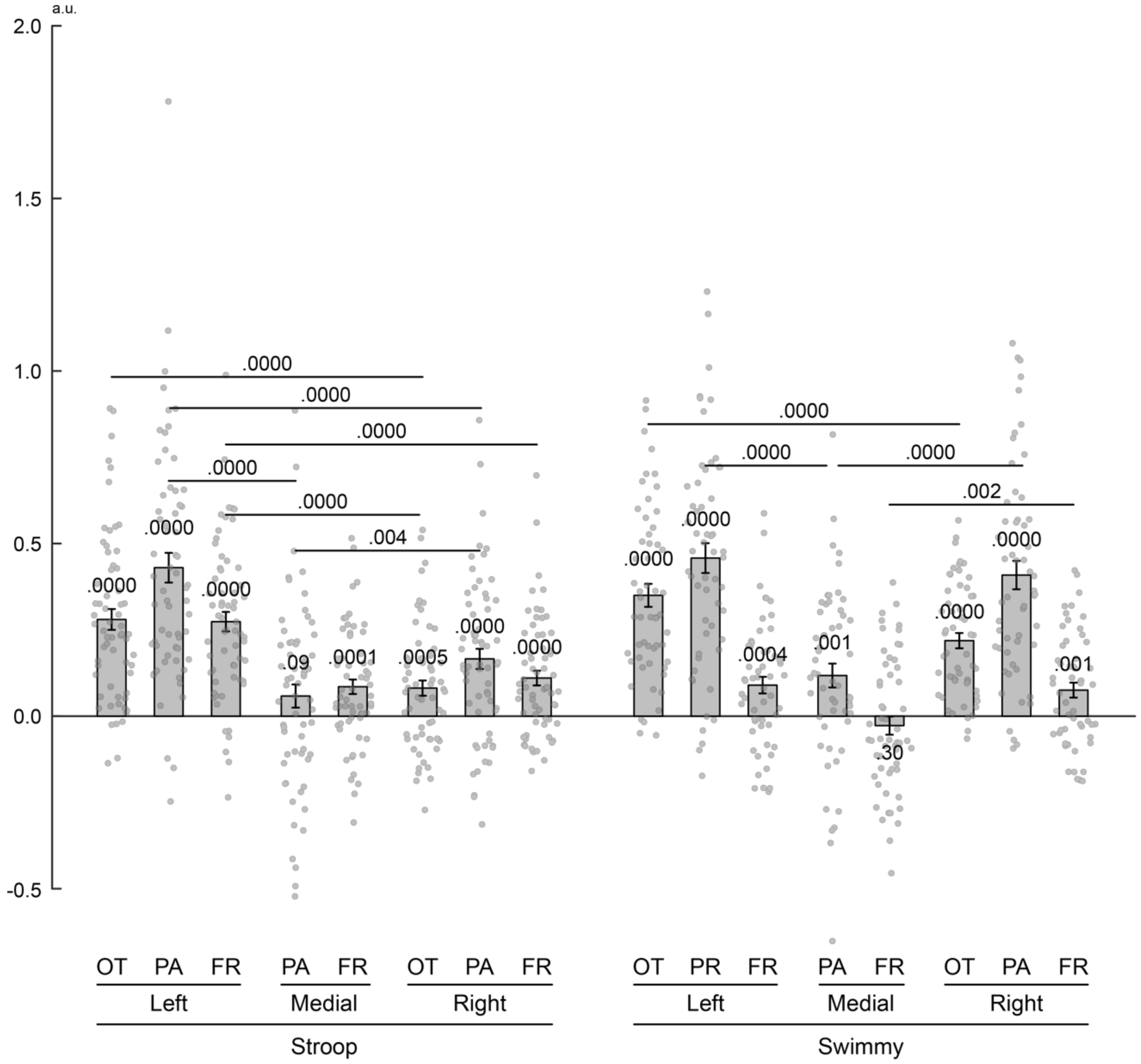

Supplementary Figure 19. Lateralized cortical involvement in the Stroop effect (Fig. 7), but not the Swimmy effect, is based in the fronto-parietal regions.
Regions of interest were defined as the occipitotemporal (OT), parietal (PA), and frontal (FR) regions in the left lateral, medial, and right lateral regions based on meta-analysis maps of cognitive control. Stroop task (*left*); and Swimmy task (*right*). Statistical procedures and formats are similar to those in Fig. 7b. The sample size is identical to that in Fig 7b.

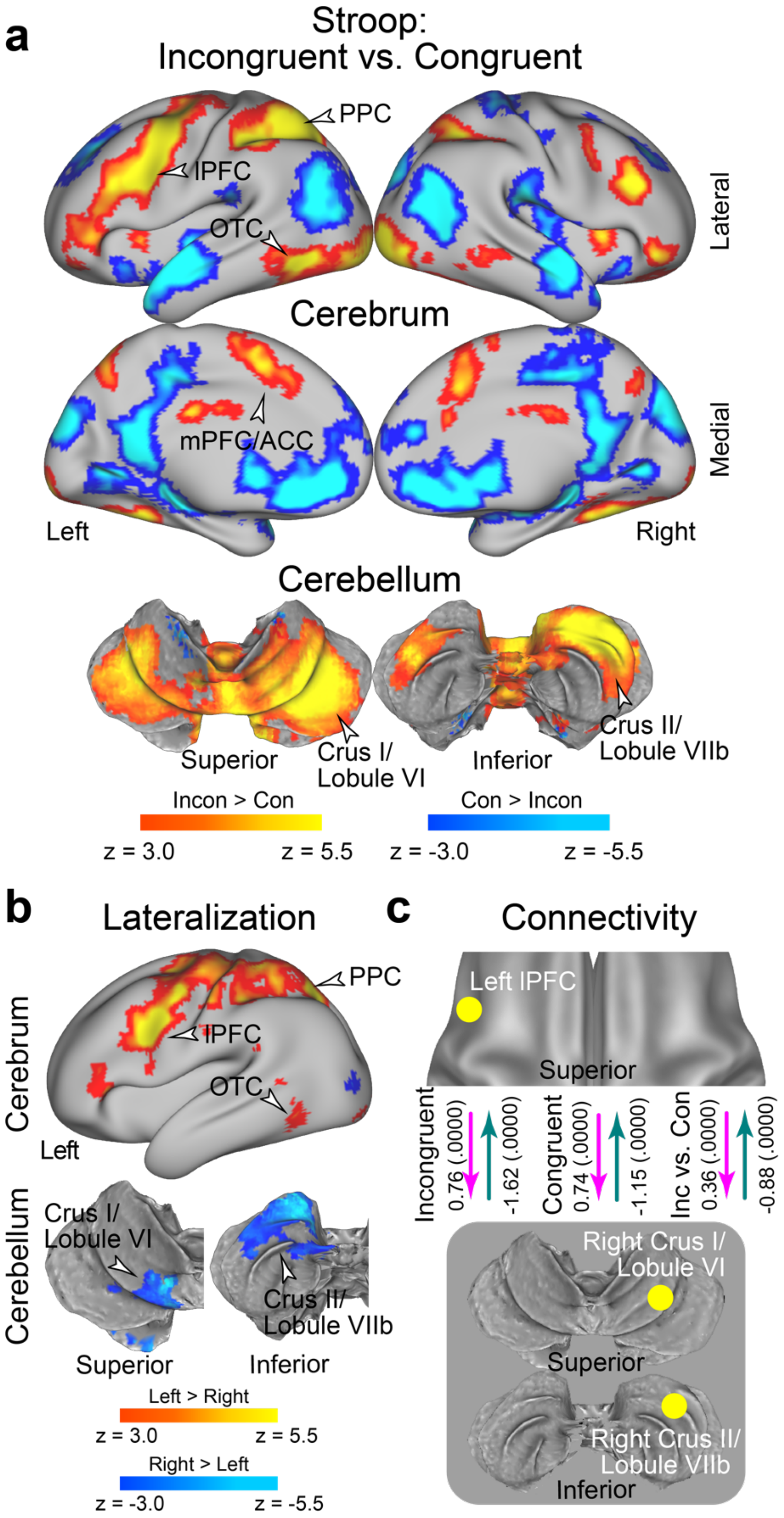

Supplementary Figure 20. Additional preprocessing of ICA-AROMA and motion censoring was administered and the imaging analyses in Fig. 3 was re-performed. **a**, Statistical activation maps. **b**, Laterality analysis. **c**, Effective connectivity analysis. Statistical procedures and formats are similar to those in Fig. 3.

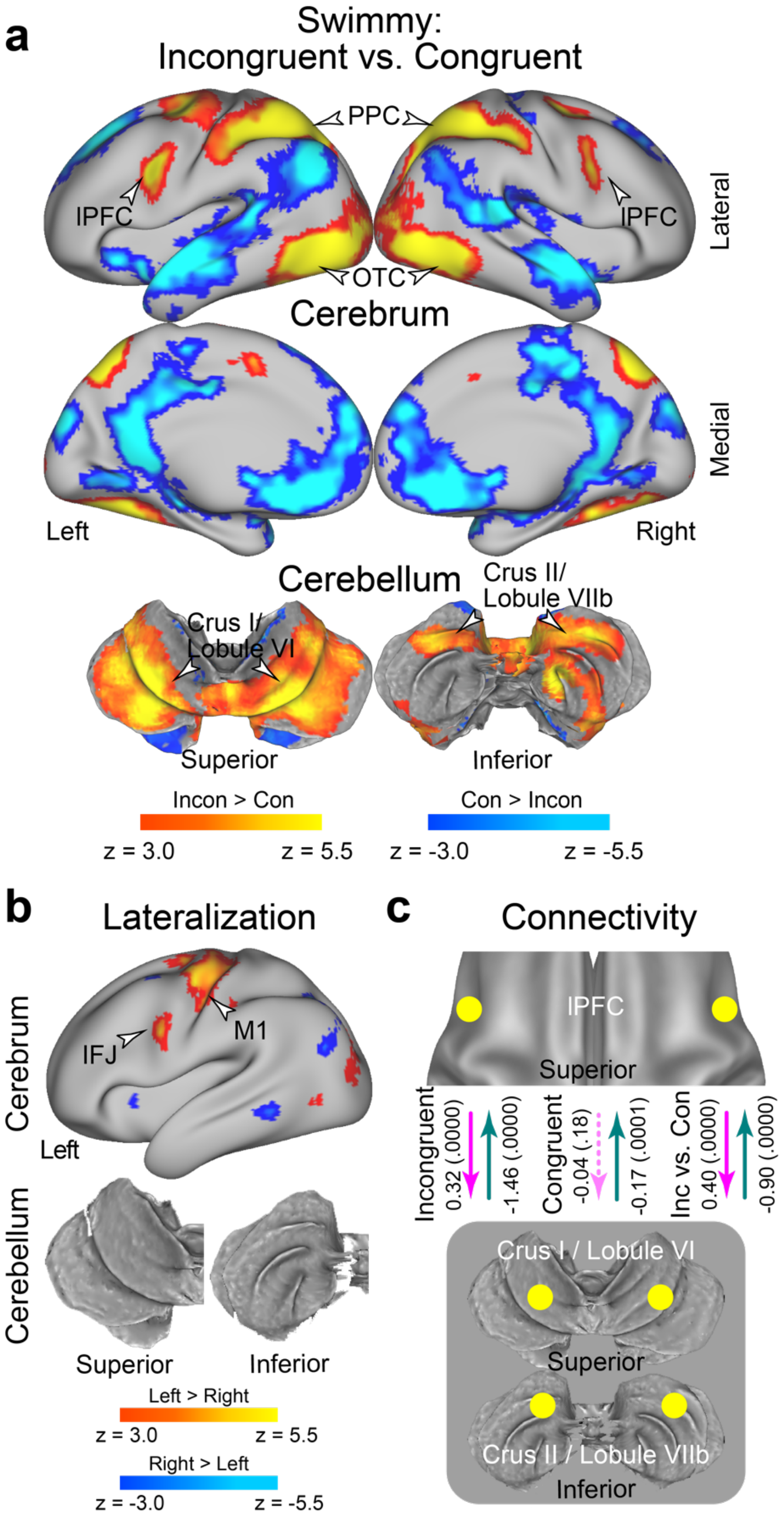

Supplementary Figure 21. Additional preprocessing of ICA-AROMA and motion censoring was administered and the imaging analyses in Fig. 4 was re-performed. **a**, Statistical activation maps. **b**, Laterality analysis. **c**, Effective connectivity analysis. Statistical procedures and formats are similar to those in Fig. 4.

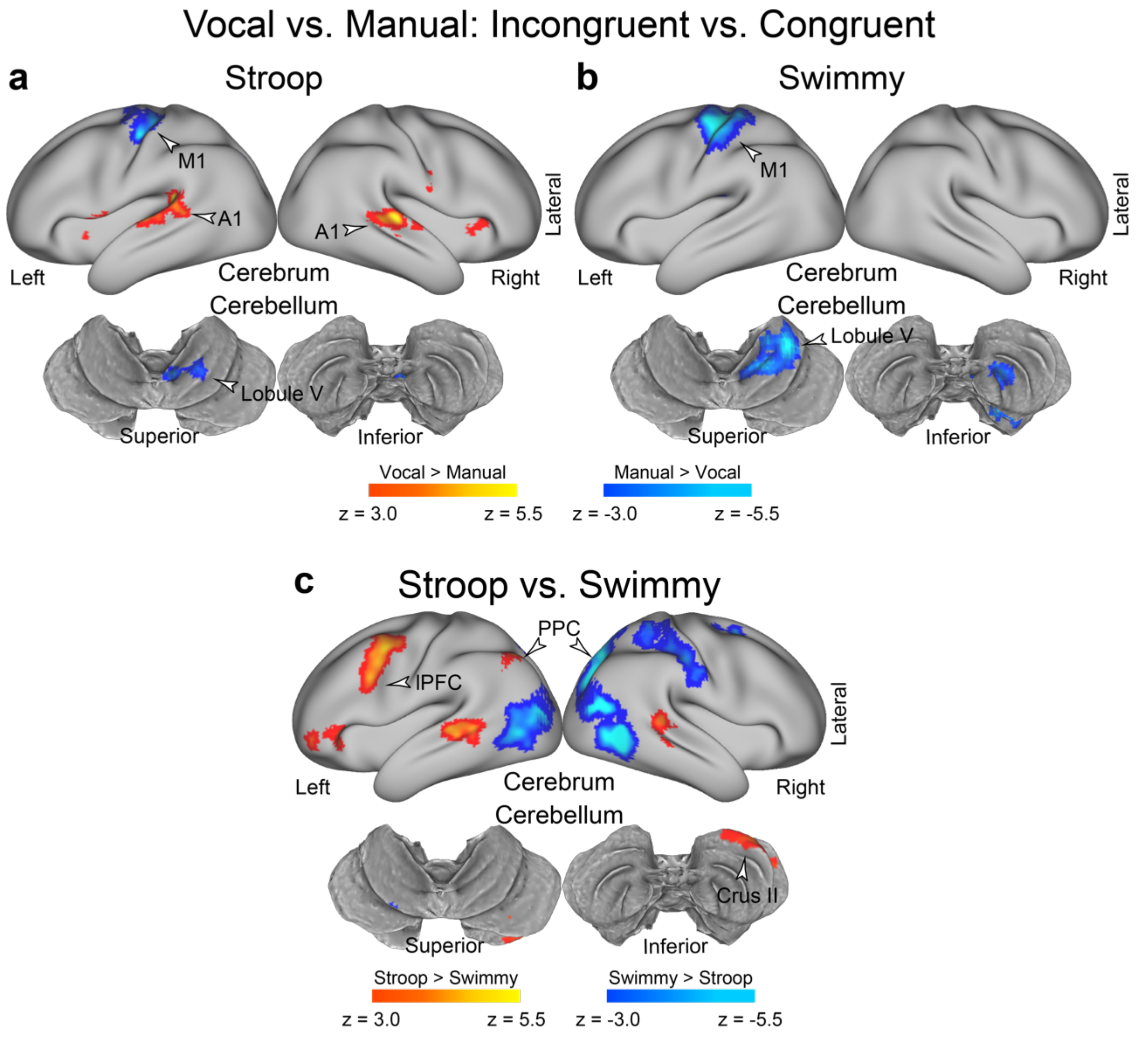

Supplementary Figure 22. Additional preprocessing of ICA-AROMA and motion censoring was administered and the imaging analyses in Figs. 5 and 6a was re-performed. Statistical maps showing a differential interference effect (incongruent vs. congruent) between the vocal and manual conditions. Stroop task (**a**); Swimmy task (**b**). **c**, Statistical maps showing a differential interference effect (incongruent vs. congruent) between the Stroop and Swimmy tasks. Formats are similar to those in Figs. 5 and 6a.

Supplementary Tables

Supplementary Table 1. Brain regions showing a significant signal increase or decrease in the contrast between incongruent and congruent trials in the Stroop task. Positive and negative z-values indicate greater signals in the incongruent and congruent trials, respectively. Coordinates are listed in MNI space. BA indicates Brodmann area and is approximate.

Area	x	y	z	z-value	BA
Frontal cortex	-50	12	32	6.74	6
	-36	14	24	6.02	44
	-42	6	54	5.99	6
	48	36	26	5.44	9
	-36	28	20	5.33	9
	-2	6	60	5.08	6
	50	46	-10	4.95	10
	4	24	42	4.70	8
	-32	-6	66	4.68	6
	24	40	-20	4.55	11
	-24	0	52	4.49	6
	44	10	44	4.49	8
	-48	42	2	4.40	46
	10	20	68	4.40	6
	-22	40	-20	4.35	11
	6	28	26	4.26	32
	8	6	68	4.03	6
	-38	50	-14	3.97	10
	48	16	14	3.52	44
	30	62	-16	3.35	10
	-50	14	0	3.33	44
	-36	42	6	3.08	46
Temporal cortex	-48	-38	46	6.50	40
	-48	-60	-12	6.38	37
	36	20	2	5.10	13
	-34	16	0	4.97	13
	-2	-26	30	4.59	23
	-4	-12	32	4.45	23
	48	-38	46	4.09	40
	-58	-50	-6	3.96	37
	56	-40	-10	3.80	21
	68	-32	-10	3.77	21
	-46	-44	-14	3.53	37
	68	-50	-6	3.26	37
	60	-56	-16	3.18	37
	56	-36	56	3.09	40
Parietal cortex	-26	-68	50	6.97	7
	-36	-56	50	6.23	39
	-28	-72	30	5.16	39
	32	-56	44	5.04	39
	-10	-70	52	4.26	7
	32	-66	62	4.03	7
	12	-68	42	3.79	7
	-32	-24	72	3.78	4

	-32	-24	58	3.51	4
Occipital cortex	-34	-86	-10	6.38	18
	30	-94	-4	6.03	18
	38	-86	-8	5.43	18
	44	-72	-10	4.23	19
Cerebellum	28	-68	-46	6.21	Lobule VIIb
	34	-64	-28	5.97	Crus I
	2	-64	-26	5.83	Vermis VI
	-32	-60	-26	5.65	Lobule VI
	0	-64	-14	5.56	Lobule V
	12	-78	-34	5.54	Crus II
	6	-78	-18	5.52	Lobule VI
	20	-66	-22	5.45	Lobule VI
	-32	-64	-46	5.39	Lobule VIIb
	-8	-80	-22	5.27	Crus I
	-18	-64	-26	4.94	Lobule VI
	0	-50	-20	4.81	Lobule I-IV
	2	-46	-6	4.80	Lobule I-IV
	-28	-76	-48	4.72	Crus II
	-44	-60	-28	4.60	Crus I
	-2	-74	-32	4.59	Crus II
	12	-62	-42	4.59	Lobule VIIIb
	14	-52	-16	4.47	Lobule V
	-10	6	2	4.08	Caudate
	36	-54	-48	3.89	Lobule VIIb
	10	-54	-30	3.45	Lobule I-IV
Others	12	2	4	4.99	Thalamus
	2	-18	-16	4.18	Thalamus
	14	-4	20	4.85	Caudate
	-12	-18	2	4.11	Thalamus
	-16	-14	22	4.10	Caudate
	8	-10	0	3.84	Thalamus
Frontal cortex	4	38	-10	-6.74	11
	2	50	-4	-6.67	10
	-22	32	36	-5.88	8
	2	8	-8	-5.86	25
	28	38	38	-5.78	9
	4	20	-8	-5.63	25
	-10	34	-10	-5.47	11
	-24	42	26	-5.25	10
	12	44	20	-4.94	9
	22	24	38	-4.89	8
	4	58	40	-4.68	9
	6	-14	50	-4.63	24
	-30	30	-16	-4.55	47
	-18	60	14	-4.51	10
	34	30	-14	-4.47	47
	-10	60	38	-4.40	9
	24	22	52	-4.33	8
	4	-26	58	-4.32	6
	58	4	2	-4.26	6
	-2	32	2	-4.24	24
	-14	40	18	-4.16	32
	-16	48	48	-4.12	8
	20	28	-8	-4.02	11
	26	52	40	-3.89	9
	22	8	58	-3.77	6

	-16	16	50	-3.68	6
	-22	34	56	-3.51	8
	-24	60	30	-3.49	10
	2	68	14	-3.44	10
	0	4	12	-3.41	24
Temporal cortex	56	-4	-14	-6.34	22
	-28	-40	-14	-6.33	37
	-50	18	-26	-6.18	38
	24	-40	-12	-6.11	37
	-60	0	-6	-6.00	22
	-6	-58	16	-5.97	23
	48	-16	22	-5.91	40
	14	-50	10	-5.88	23
	-52	-8	-18	-5.86	21
	32	-32	-16	-5.57	36
	48	18	-28	-5.52	38
	2	-46	22	-4.91	23
	38	-6	2	-4.88	13
	12	-52	28	-4.81	23
	62	-22	22	-4.78	40
	-44	-10	-2	-4.72	13
	-10	-50	28	-4.70	23
	38	2	16	-4.63	13
	-36	-18	4	-4.27	13
	-18	-32	-14	-4.13	36
	34	18	-28	-4.12	38
	-54	-28	12	-4.11	41
	34	-18	18	-4.03	13
	40	10	-16	-3.98	13
	16	-22	32	-3.66	23
	48	4	-8	-3.55	13
	40	-44	0	-3.47	37
	-50	-20	20	-3.21	40
	-30	-14	16	-3.19	13
Parietal cortex	-52	-76	24	-5.58	39
	4	-38	56	-5.42	5
	28	-36	62	-5.20	1
	-44	-80	34	-4.95	39
	36	-18	46	-4.81	4
	-12	-34	42	-4.76	31
	40	-48	24	-4.03	39
	48	-12	52	-3.99	4
	6	-28	76	-3.98	4
	10	-40	80	-3.96	1
	18	-30	72	-3.96	4
	-36	-22	24	-3.91	1
	40	-30	62	-3.86	1
	-12	-34	56	-3.72	5
	-24	-22	42	-3.69	1
	2	-48	68	-3.68	7
	58	-10	38	-3.50	4
	-68	-20	22	-3.48	1
	36	-30	42	-3.48	1
	12	-52	62	-3.41	7
	-16	-86	48	-3.15	7
Occipital cortex	52	-64	12	-6.04	19
	-14	-48	2	-5.59	18

	12	-86	36	-5.56	19
	-12	-98	22	-5.55	18
	-42	-72	8	-5.48	19
	18	-92	24	-5.16	18
	-20	-68	-2	-5.14	19
	24	-68	0	-5.04	19
	-26	-56	-6	-4.94	19
	30	-50	-4	-4.83	19
	0	-88	26	-4.74	18
	10	-74	2	-4.70	18
	-44	-84	14	-4.61	19
	18	-84	48	-4.32	19
	20	-68	26	-3.58	19
	-54	-78	4	-3.45	19
	-4	-76	26	-3.38	18
Others	22	-16	-18	-7.00	Hippocampus
	-24	-20	-14	-6.39	Hippocampus
	28	8	-20	-4.68	Amygdala
	-30	0	-22	-4.53	Amygdala
	32	-4	-18	-4.43	Hippocampus
	-16	0	-22	-3.46	Amygdala

Supplementary Table 2. Brain regions showing a significant signal increase or decrease in the contrast between incongruent and congruent trials in the Swimmy task. Formats are similar to those in Supplementary Table 1.

Area	x	y	z	z-value	BA
Frontal cortex	-24	-8	50	5.94	6
	-46	6	34	5.49	6
	26	0	54	5.42	6
	-36	-6	66	5.39	6
	-52	8	22	4.64	44
	54	14	36	4.59	44
	42	6	30	4.27	6
	-28	-18	74	3.43	6
	-10	-6	56	3.24	6
Temporal cortex	46	-62	-12	7.91	37
	-46	-64	-6	7.06	37
	-36	-42	42	6.78	40
	48	-38	56	6.12	40
	34	-74	-14	5.78	37
	-42	-50	-14	5.09	37
Parietal cortex	14	-68	58	7.26	7
	-18	-72	56	6.92	7
	28	-58	50	6.81	7
	-24	-72	36	6.57	7
	30	-70	34	6.51	7
	-28	-58	42	6.45	7
	38	-46	54	6.24	7
	-38	-48	54	6.09	7
	-28	-62	60	6.00	7
	-54	-16	52	3.73	1
	-38	-18	58	3.34	4
Occipital cortex	-34	-88	4	7.00	18
	-42	-78	-6	6.90	19
	-32	-86	18	6.66	19
	34	-84	12	6.17	19
Cerebellum	20	-68	-22	6.22	Lobule VI
	16	-56	-44	6.13	Lobule IX
	-30	-58	-24	5.73	Lobule VI
	-18	-68	-24	5.72	Lobule VI
	-42	-58	-26	5.38	Crus I
	6	-78	-28	5.34	Crus II
	2	-66	-18	5.32	Vermis VI
	28	-74	-48	5.14	Crus II
	-32	-40	-34	5.11	Lobule VI
	-6	-78	-26	5.00	Crus I
	-26	-70	-46	4.79	Lobule VIIb
	38	-66	-24	4.78	Crus I
	16	-76	-42	4.76	Crus II
	4	-64	-36	4.75	Vermis VIIIa
	34	-48	-26	4.72	Lobule VI
	-16	-42	-44	4.45	Lobule X
	30	-42	-46	4.45	Lobule VIIIa
	28	-58	-32	4.43	Lobule VI
	-14	-54	-46	4.17	Lobule IX
	16	-64	-54	4.11	Lobule VIIIa

	-28	-54	-44	4.02	Lobule VIIIa
	30	-56	-50	3.87	Lobule VIIIa
	-28	-42	-54	3.74	Lobule VIIIa
Frontal cortex	0	40	-8	-6.45	11
	-28	34	46	-6.26	8
	6	24	-12	-5.93	11
	12	52	14	-5.32	10
	2	52	-8	-5.27	10
	-2	56	28	-5.27	9
	2	10	-14	-5.26	25
	12	38	-8	-5.24	11
	-10	26	-10	-5.22	32
	12	48	26	-5.15	9
	0	-16	44	-5.10	24
	36	4	-18	-4.86	34
	0	56	14	-4.77	10
	-10	28	64	-4.76	6
	28	40	40	-4.74	9
	12	-12	44	-4.69	24
	-14	34	38	-4.66	8
	-16	44	50	-4.66	8
	-36	30	-12	-4.65	47
	50	-6	8	-4.58	6
	6	-14	62	-4.47	6
	0	50	46	-4.38	9
	8	-18	74	-4.35	6
	-4	40	58	-4.18	8
	-22	50	28	-4.13	10
	-10	42	8	-4.10	32
	24	30	60	-4.10	8
	0	32	4	-4.09	24
	-16	56	12	-3.94	10
	-8	66	18	-3.92	10
	-14	36	20	-3.78	32
	-22	22	-14	-3.60	11
	10	66	6	-3.29	10
	-48	26	-2	-3.25	45
	12	28	50	-3.13	8
Temporal cortex	68	-32	14	-6.23	22
	56	-4	-20	-5.87	21
	-8	-48	4	-5.78	30
	60	4	-12	-5.77	38
	-22	-20	-14	-5.69	36
	-40	16	-22	-5.69	38
	10	-54	12	-5.54	23
	-60	-10	-14	-5.32	21
	-58	2	-16	-5.22	38
	36	18	-26	-5.21	38
	46	-16	-4	-5.19	22
	-68	-40	14	-4.99	22
	-12	-30	-6	-4.91	36
	-28	-32	-18	-4.85	37
	-28	10	-20	-4.84	13
	50	22	-18	-4.84	38
	-56	-38	18	-4.76	22
	-42	18	-34	-4.69	38
	-50	14	-14	-4.68	38

	48	-2	-32	-4.56	20
	-42	-22	0	-4.45	41
	-56	4	-4	-4.39	22
	22	-32	-16	-4.26	36
	-68	-32	2	-4.25	21
	38	-26	10	-3.87	41
	12	-50	28	-3.87	23
	-68	-48	2	-3.84	21
	58	-20	-12	-3.79	21
	-24	16	-36	-3.74	38
	-52	-18	-22	-3.70	20
	-40	-4	-14	-3.69	22
	-58	-38	-2	-3.58	21
	-44	-4	-2	-3.58	13
	-44	0	-32	-3.30	38
Parietal cortex	66	-46	14	-5.96	39
	4	-28	60	-5.60	4
	-50	-70	36	-5.36	39
	34	-18	48	-5.30	4
	-2	-32	48	-5.25	31
	0	-44	40	-5.22	31
	38	-22	22	-4.90	1
	-4	-42	56	-4.87	31
	46	-34	22	-4.85	40
	-64	-26	16	-4.75	40
	50	-52	18	-4.69	39
	-6	-54	30	-4.63	31
	-64	-56	16	-4.37	39
	52	-68	42	-4.36	39
	10	-30	74	-4.31	4
	24	-32	64	-4.07	4
	-58	-66	20	-3.98	39
	50	-18	14	-3.97	40
	60	-62	28	-3.97	39
	36	-28	64	-3.96	4
	48	-12	54	-3.85	4
	-36	-20	18	-3.79	1
	64	-52	34	-3.73	39
	-10	-32	76	-3.47	4
	44	-64	34	-3.19	39
Occipital cortex	-8	-94	30	-5.44	18
	14	-46	2	-5.37	18
	16	-92	38	-5.12	19
	6	-86	32	-5.07	19
	-2	-68	22	-4.75	18
	-14	-66	-6	-4.43	19
	-6	-102	18	-4.25	18
	60	-66	8	-4.21	19
	6	-66	2	-4.21	18
	-14	-58	20	-4.15	18
	-14	-42	-8	-3.98	19
	-10	-78	-6	-3.89	18
	22	-56	-4	-3.71	19
	56	-70	20	-3.24	19
	-10	-80	28	-3.19	18
	-32	-52	-4	-3.19	19
	-10	-90	42	-3.03	19

Cerebellum	16	-92	-34	-4.93	Crus II
	-20	-92	-34	-4.26	Crus II
	34	-86	-34	-3.71	Crus II
	-32	-80	-34	-3.48	Crus II
	-20	-78	-34	-3.26	Crus II
	-46	-76	-36	-3.26	Crus I
Others	24	-16	-16	-5.45	Hippocampus
	-22	0	-4	-4.62	Pallidum
	20	4	-18	-4.45	Amygdala
	-32	-38	-6	-4.11	Hippocampus
	-18	0	-16	-4.04	Amygdala
	10	-26	4	-3.72	Thalamus
	20	-6	-6	-3.63	Pallidum

Supplementary Table 3. Brain regions showing a significant interaction effect of response modality (vocal and manual) and congruency (incongruent and congruent) in the Stroop task. Formats are similar to those in Supplementary Table 1.

Area	x	y	z	z-value	BA
Frontal cortex	0	28	64	4.27	6
	18	46	24	4.12	9
	12	60	24	4.11	9
	-58	12	0	3.90	44
	12	24	62	3.74	6
	32	34	24	3.64	9
	46	24	-4	3.63	47
	34	46	20	3.43	10
	0	52	26	3.37	9
	-10	42	34	3.31	9
	-44	20	-6	3.30	47
Temporal cortex	-50	-40	18	4.82	22
	64	-28	6	4.59	22
	-60	-20	6	4.16	41
	58	-10	0	4.01	41
	-62	-8	6	3.69	41
	-64	-40	10	3.67	22
	54	14	-8	3.67	38
	48	-30	2	3.33	22
	68	-12	8	3.20	41
Parietal cortex	-64	-42	22	3.16	39
Frontal cortex	32	-8	54	-4.11	6
	-10	-2	56	-4.07	6
	-18	-10	70	-3.94	6
	-4	-14	54	-3.55	6
Temporal cortex	40	-42	-22	-3.58	37
Parietal cortex	-32	-30	52	-4.93	4
	-40	-14	54	-4.68	4
	-8	-60	58	-4.67	7
	-34	-78	40	-4.27	39
	6	-58	50	-4.16	7
	10	-64	60	-3.07	7
Occipital cortex	-34	-72	20	-3.96	19
	-18	-66	26	-3.73	19
Cerebellum	8	-54	-14	-4.53	Lobule V
	40	-42	-34	-3.95	Lobule VI
	24	-42	-20	-3.81	Lobule V
	18	-46	-22	-3.23	Lobule V

Supplementary Table 4. Brain regions showing a significant interaction effect of response modality (vocal and manual) and congruency (incongruent and congruent) in the Swimmy task. Formats are similar to those in Supplementary Table 1.

Area	x	y	z	z-value	BA
Frontal cortex	-40	-16	68	-4.83	6
	-4	-12	56	-4.16	6
	-4	-2	48	-4.10	6
	-26	-22	76	-4.08	6
	-14	-16	70	-3.72	6
Temporal cortex	-44	-26	24	-4.30	40
Parietal cortex	-32	-28	62	-5.18	4
	-32	-26	50	-5.15	4
	-46	-22	54	-4.70	1
	-30	-40	64	-4.04	1
Cerebellum	26	-44	-28	-5.25	Lobule VI/V
	18	-52	-18	-3.93	Lobule V

Supplementary Table 5. Brain regions showing differential brain activity in the interference effects (incongruent vs. congruent) between the Stroop and Swimmy tasks. Formats are similar to those in Table 1. *: P < .05 FWE-corrected across the whole cerebellum based on non-parametric permutation tests.

Area	x	y	z	z-value	BA
Frontal cortex	-38	16	30	4.56	44
	-44	8	52	4.37	6
	-48	14	42	4.18	8
	-40	40	-8	4.04	47
	-2	26	48	3.91	8
	-44	14	12	3.90	44
	-36	30	22	3.57	9
	-42	54	-6	3.55	10
	-2	14	58	3.49	6
	2	32	30	3.34	8
	-50	36	12	3.31	46
	10	24	26	3.31	32
	10	26	64	3.28	6
Temporal cortex	-64	-30	0	4.04	21
	-58	-46	0	3.72	21
	52	-32	-8	3.64	21
	54	-38	8	3.61	22
	72	-34	0	3.56	21
Parietal cortex	-40	-72	50	3.83	39
	-58	-60	36	3.13	39
Cerebellum	34	-74	-42	3.41*	Crus II
	44	-80	-38	3.39*	Crus I
Frontal cortex	22	6	56	-4.33	6
	24	-8	54	-3.53	6
	28	0	68	-3.24	6
Temporal cortex	54	-64	-2	-5.40	37
Parietal cortex	32	-82	34	-5.31	39
	28	-70	44	-4.96	7
	10	-62	64	-4.47	7
	44	-26	36	-4.24	1
	56	-22	46	-3.94	1
	30	-42	48	-3.91	7
	46	-34	62	-3.87	1
	60	-12	38	-3.83	4
	32	-46	68	-3.73	1
	24	-58	46	-3.71	7
	-16	-76	56	-3.51	7
	-24	-82	44	-3.23	7
Occipital cortex	46	-76	18	-5.78	19
	-40	-86	14	-5.38	19
	-52	-74	-2	-4.42	19
	-44	-70	8	-4.32	19
	-26	-70	-12	-4.03	19
	-26	-58	-8	-3.80	19
	-22	-80	18	-3.22	19
