# Supplementary material for: The Stroop effect involves an excitatory–inhibitory fronto-cerebellar loop
Source: Nat Commun. 2023 Jan 11;14:27. doi: 10.1038/s41467-022-35397-w (PMC9834394; doi:10.1038/s41467-022-35397-w)
Supplement: Supplementary file 3 — Reporting Summary [file 41467_2022_35397_MOESM3_ESM.pdf]

nature research

Corresponding author(s): Koji Jimura

Last updated by author(s): Nov 18, 2022

Reporting Summary

Nature Research wishes to improve the reproducibility of the work that we publish. This form provides structure for consistency and transparency in reporting. For further information on Nature Research policies, see our Editorial Policies and the Editorial Policy Checklist.

Statistics

For all statistical analyses, confirm that the following items are present in the figure legend, table legend, main text, or Methods section.

n/a	Confirmed	
☐	☒	The exact sample size (*n*) for each experimental group/condition, given as a discrete number and unit of measurement
☐	☒	A statement on whether measurements were taken from distinct samples or whether the same sample was measured repeatedly
☐	☒	The statistical test(s) used AND whether they are one- or two-sided<br>*Only common tests should be described solely by name; describe more complex techniques in the Methods section.*
☐	☒	A description of all covariates tested
☐	☒	A description of any assumptions or corrections, such as tests of normality and adjustment for multiple comparisons
☐	☒	A full description of the statistical parameters including central tendency (e.g. means) or other basic estimates (e.g. regression coefficient) AND variation (e.g. standard deviation) or associated estimates of uncertainty (e.g. confidence intervals)
☐	☒	For null hypothesis testing, the test statistic (e.g. *F*, *t*, *r*) with confidence intervals, effect sizes, degrees of freedom and *P* value noted<br>*Give P values as exact values whenever suitable.*
☐	☒	For Bayesian analysis, information on the choice of priors and Markov chain Monte Carlo settings
☐	☒	For hierarchical and complex designs, identification of the appropriate level for tests and full reporting of outcomes
☐	☒	Estimates of effect sizes (e.g. Cohen's *d*, Pearson's *r*), indicating how they were calculated

Our web collection on statistics for biologists contains articles on many of the points above.

Software and code

Policy information about availability of computer code

Data collection	Behavioral data was collected via E-prime programs (Psychology software Tools, Inc.; ver. 2.0.10.356). Functional Resonance Imaging Data (fMRI) were acquired using a whole-body 3T MRI system (Siemens Prisma).
Data analysis	Data were analyzed using Matlab 2017a, SPSS Statistics (ver. 25), SPM12 (ver. 6685), SUIT (Diedrichsen & Zotow, 2015; ver. 3.4), FSL (ver. 5.0.9), Connectome Workbench (ver. 1.4.2).

For manuscripts utilizing custom algorithms or software that are central to the research but not yet described in published literature, software must be made available to editors and reviewers. We strongly encourage code deposition in a community repository (e.g. GitHub). See the Nature Research guidelines for submitting code & software for further information.

Data

Policy information about availability of data

All manuscripts must include a data availability statement. This statement should provide the following information, where applicable:
- Accession codes, unique identifiers, or web links for publicly available datasets
- A list of figures that have associated raw data
- A description of any restrictions on data availability

The neuroimaging timeseries and relevant event-onset data generated in this study have been deposited in the Dryad database (https://doi.org/10.5061/dryad.msbcc2g2p). The behavioral and ROI data generated in this study are provided in the Source Data file. The database of Neurosynth is publicly available (https://neurosynth.org/).

Field-specific reporting

Please select the one below that is the best fit for your research. If you are not sure, read the appropriate sections before making your selection.

☒ Life sciences ☐ Behavioural & social sciences ☐ Ecological, evolutionary & environmental sciences

For a reference copy of the document with all sections, see nature.com/documents/nr-reporting-summary-flat.pdf

Life sciences study design

All studies must disclose on these points even when the disclosure is negative.

Sample size	Sample size was determined before the collection of the data based on behavioral and neuroimaging pilot experiments.
Data exclusions	One participant in one experimental condition (vocal Stroop) was excluded from analyses due to low behavioral performance. In other analyses, no data were excluded.
Replication	For each participant, one experiment was performed independently, and therefore, a total of 119 independent experiments were performed. Statistical tests were performed by multiple designs and models, and all fundamental results were replicated.
Randomization	Order of experimental conditions were randomized for each participant.
Blinding	Group allocation was not blind to investigators and experimenters because each participant was assigned to one of the four experimental conditions in which behavioral tasks were performed using distinct visual stimulus sets and response types.

Reporting for specific materials, systems and methods

We require information from authors about some types of materials, experimental systems and methods used in many studies. Here, indicate whether each material, system or method listed is relevant to your study. If you are not sure if a list item applies to your research, read the appropriate section before selecting a response.

Materials & experimental systems

n/a	Involved in the study	
☒	☐	Antibodies
☒	☐	Eukaryotic cell lines
☒	☐	Palaeontology and archaeology
☒	☐	Animals and other organisms
☐	☒	Human research participants
☒	☐	Clinical data
☒	☐	Dual use research of concern

Methods

n/a	Involved in the study	
☒	☐	ChIP-seq
☒	☐	Flow cytometry
☐	☒	MRI-based neuroimaging

Human research participants

Policy information about studies involving human research participants

Population characteristics	Healthy young human participants (N = 119; age range: 18-24; 35 female) were right handed and had no history of psychiatric or neurological disorders.
Recruitment	An advertisement recruited participants at the Kochi University of Technology. Self-selection bias may be involved in the results of the current study potentially; its effect may be equivalent to that in standard neuroimaging studies where healthy young human volunteers participated.
Ethics oversight	All experimental procedures were approved by the institutional review boards of Keio University and Kochi University of Technology.

Note that full information on the approval of the study protocol must also be provided in the manuscript.

Magnetic resonance imaging

Experimental design

Design type	Participants performed a behavioral task involving four trial conditions while being scanned. An event-related fMRI design was used.

Design specifications	Participants performed 5 runs during fMRI scanning. Each run involved 48 trials with 12 trials for each of the four trial conditions. One functional run lasted 192 sec with 277 volume acquisition. Duration of the stimulus presentation was 2 sec followed by 2-sec ITI.
Behavioral performance measures	We recorded vocal responses and button presses to analyze behavioral performance.

Acquisition

Imaging type(s)	Functional and structural images of the human brain were collected.
Field strength	3 Tesla
Sequence & imaging parameters	Functional MRI scanning was conducted on a whole-body 3T MRI system (Siemens Prisma, Germany). Functional images were acquired using multi-band accelerated gradient-echo echo-planar imaging [repetition time (TR) = 743 ms; echo time (TE) = 35.6 ms; flip angle (FA) = 48 degrees; slice thickness, 2 mm; in-plane resolution, 2 x 2 mm; multi-band factor (MBF) = 8; 72 slices]. High-resolution anatomical images were acquired using an MP-RAGE T1-weighted sequence [TR = 1900 msec; TE = 2.52 msec; FA: 9 deg; 176 slices; slice thickness, 1 mm; in-plane resolution, 1 x 1 mm2].
Area of acquisition	A whole-brain scan was used.

Diffusion MRI ☐ Used ☒ Not used

Preprocessing

Preprocessing software	Imaging data was analyzed using SPM12 (http://www.fil.ion.ucl.ac.uk/spm/). All functional images were first temporally aligned across the brain volume, corrected for movement using a rigid-body rotation and translation correction, and then registered to the participant's anatomical images to correct for movement between the anatomical and function scans. The functional images were then registered to the reference brain using the alignment parameters derived for the anatomical scans. The data were then resampled into 2 mm isotropic voxels, and spatially smoothed with a 6 mm full-width at half-maximum Gaussian kernel.
Normalization	Functional images were first co-registered to an individuals' high-resolution structural image and then registered to a standardized MNI template using SPM12. For the analysis dedicated to the cerebellum, cerebellar images were registered to a standard SUIT template using SUIT/SPM12 (http://www.diedrichsenlab.org/imaging/suit.htm).
Normalization template	A standardized template (MNI152) was used for the whole-brain analysis. Another standardized template (SUIT) was used for the cerebellar analysis.
Noise and artifact removal	In order to minimize motion-derived artifacts due to consumption of liquid rewards, functional images were further preprocessed by general linear model (GLM) estimations with motion parameters and MRI signal time courses (cerebrospinal fluid, white matter, and whole brain), and their derivatives and quadratics as nuisance regressors based on fsl_regfilt implemented in the FSL suite (http://www.fmrib.ox.ac.uk/; ver. 5.0.9). Then, the residual of this nuisance GLM was used in the analysis. For supplementary analysis, additional preprocessing was applied using ICA-AROMA and motion censoring (Power et al. 2012).
Volume censoring	The initial 10 volumes of each run were excluded from imaging analysis to take into account the equilibrium of longitudinal magnetization.

Statistical modeling & inference

Model type and settings	A GLM approach was used to estimate event-related activity for each participant. In single-level analysis, SPM was used. In group-level analysis, maps of beta estimate contrasts were collected from all samples, and group-mean tests were performed based on permutation methods using ramdomise in the FSL suite.
Effect(s) tested	We used a 2-by-2 factorial design with two levels of stimulus type (Stroop, Swimmy) and two levels of response type (vocal, manual). Each of the four conditions (e.g., vocal Stroop) involved two levels of trial type (e.g., word naming, color naming), and two levels of stimulus congruency (incongruent, congruent), entailing a 2 x 2 factorial design.

Specify type of analysis: ☐ Whole brain ☐ ROI-based ☒ Both

Anatomical location(s)	In functional connectivity analyses, ROIs were defined based on activation maps of other experimental conditions, independently of tested data, in order to avoid circular analysis. In univariate activation analyses, ROIs were defined based on meta-analysis maps in Neurosynth (https://neurosynth.org/)
Statistic type for inference (See Eklund et al. 2016)	In exploratory analyses, group-mean tests were performed based on non-parametric permutation methods implemented by randomize in the FSL suite. Voxel clusters were identified using a voxel-wise uncorrected threshold of P < .001. This procedure was validated to appropriately control the false-positive rate in Eklund et al. 2016.
Correction	The voxel clusters were tested for a significance using a threshold of P < .05 corrected by family-wise error rate.

Models & analysis

n/a	Involved in the study
☐	☒ Functional and/or effective connectivity
☒	☐ Graph analysis
☒	☐ Multivariate modeling or predictive analysis

Functional and/or effective connectivity	MRI timeseries of the lateral prefrontal cortex and cerebellum were subjected to dynamic causal modeling analysis (Friston et al. 2003) to estimate model parameters reflecting task-related directional functional connectivity.
